# The Five Diamond Method for Explorative Business Process Management

**DOI:** 10.1007/s12599-021-00703-1

**Published:** 2021-06-08

**Authors:** Thomas Grisold, Steven Groß, Katharina Stelzl, Jan vom Brocke, Jan Mendling, Maximilian Röglinger, Michael Rosemann

**Affiliations:** 1 University of Liechtenstein, Vaduz, Liechtenstein; 2 Vienna University of Economics and Business, Vienna, Austria; 3 University of Bayreuth, Bayreuth, Germany; 4 Queensland University of Technology, Brisbane, Australia

**Keywords:** Business process management, Explorative BPM, Ambidextrous BPM, Innovation management, Digital technologies, Design science research, Situational method engineering

## Abstract

Explorative business process management (BPM) is attracting increasing interest in the literature and professional practice. Organizations have recognized that a focus on operational efficiency is no longer sufficient when disruptive forces can make the value proposition of entire processes obsolete. So far, however, research on how to create entirely new processes has remained largely conceptual, leaving it open how explorative BPM can be put into practice. Following the design science research paradigm and situational method engineering, we address this research gap by proposing a method called the Five Diamond Method. This method guides explorative BPM activities by supporting organizations in identifying opportunities from business and technology trends and integrating them into business processes with novel value propositions. The method is evaluated against literature-backed design objectives and competing artifacts, qualitative data gathered from BPM practitioners, as well as a pilot study and two real-world applications. This research provides two contributions. First, the Five Diamond Method broadens the scope of BPM by integrating prescriptive knowledge from innovation management. Second, the method supports capturing emerging opportunities arising from changing customer needs and digital technologies.

**Supplementary Information:**

The online version contains supplementary material available at 10.1007/s12599-021-00703-1.

## Introduction

Business process management (BPM) helps organizations operate in an effective and efficient way through the continuous discovery, execution, analysis, and redesign of business processes (Dumas et al. 2018). To this end, an extensive set of BPM methods and tools help achieve stability, efficiency, and effectiveness (Gross et al. 2019; Rosemann 2014; vom Brocke et al. 2020) by building on different redesign rationales (Gross et al. in press). In analogy to the concept of organizational ambidexterity (O'Reilly and Tushman 2013), these approaches can be largely classified as being *exploitative.* They tend to neglect new innovation opportunities (Benner and Tushman 2003; Berente and Lee 2014). Little attention has been payed to *explorative* BPM methods for proactively integrating opportunities into business processes with novel value propositions (Grisold et al. 2019; Rosemann 2014, 2020).

We see more and more claims emerging in the literature which stress the importance of extending BPM with more innovation-oriented concepts. A key idea of *explorative* BPM is to ensure that organizations systematically integrate emerging opportunities, such as those brought about by digital technologies or changing customer needs, in order to offer new value propositions (Beverungen et al. 2020; Grisold et al. 2019; Kerpedzhiev et al. 2021; Rosemann 2014). This is important in today’s rapidly evolving business environment. Digital ecosystems (e.g., Apple Store), platform-based subscription models (e.g., Netflix), or GPS-based location of customers (e.g., Uber) are just a few examples of how digital technologies have changed how organizations operate, interact with customers, and create revenue models. While early BPM approaches proposed to capitalize on such developments to radically innovate business processes (Hammer and Champy 1994; Kettinger et al. 1997), most of today’s tools and methods lack a focus on exploration (Gross et al. 2019; Rosemann 2014; vom Brocke et al. 2020). This is to the detriment of organizations that struggle with realizing the potential of exploring novel business processes and securing success in dynamic business environments (Rosemann 2020).

Against this backdrop, we address the following research question: *How can we realize explorative BPM to systematically identify new value propositions for business processes?* To answer this question, we developed a BPM method which aims at enabling explorative BPM in organizations: the *Five Diamond Method*. In doing so, we adopted the design science research (DSR) paradigm (Gregor and Hevner 2013). Our key conceptual move is that we deliberately integrate and synthesize approaches from the innovation management (IM) literature to enhance the innovation focus of our BPM method. As a result, we present a BPM method that supports organizations to (1) identify innovation opportunities resulting from digital technologies and changing customer needs, and (2) integrate these opportunities into new business processes with novel value propositions. We evaluated the Five Diamond Method in a variety of ways. We found that it provides a comprehensive explorative BPM approach, helping practitioners benefit from emerging business and technology opportunities. In particular, the evaluations showed that our method supported the generation of process-related ideas that were perceived as fundamentally new in relation to existing processes. Furthermore, we found that our method helps to systematically uncover key trends and develop explorative BPM ideas in a relatively short time. In principle, the Five Diamond Method can be applied in different organizational contexts. However, it presupposes that the organization has a well-developed process orientation, thus making it particularly relevant for medium-sized and large organizations. From an academic perspective, we argue that this constitutes an important contribution to the existing BPM literature which has to date primarily focused on the enhancement of operational efficiency, i.e., exploitation (Gross et al. 2019; Rosemann 2014; vom Brocke et al. 2020).

We will proceed as follows: Sect. 2 discusses the theoretical background of BPM and IM. Section 3 outlines our research method, while Sect. 4 specifies the Five Diamond Method and Sect. 5 reports on its evaluation. We derive implications and limitations in Sect. 6.

## Theoretical Background

### Explorative Business Process Management

Business process management (BPM) comprises principles, methods, techniques, and tools to discover, execute, analyze, redesign, and monitor business processes (Dumas et al. 2018). It aims to maintain a business process focus within the management of work in organizations (Dumas et al. 2018). BPM as a discipline emerged from the Business Process Reengineering (BPR) management concept in the 1990s, which aimed for the radical rethinking of existing business processes to achieve significant increases in performance (Hammer and Champy 1994). Various BPR projects transformed working routines and organizational practices (Ozcelik 2010). This was primarily driven by the fact that emerging technologies enabled new means to carry out work (Hammer and Champy 1994; Kettinger et al. 1997). In this respect, BPR’s ambition was to fundamentally rethink how work is done (Hammer 1990). Rather than following the radical ambition of BPR, most subsequent BPM approaches gravitated towards the idea of continuous yet incremental changes of existing processes (Gross et al. 2019; Rosemann 2014; vom Brocke et al. 2020). Methodologies such as lean management or Six Sigma are popular examples as they had a substantial impact on the operational performance of organizations around the globe.

While process change can be incremental (i.e., small variations to the process design) or radical (i.e., an entirely new process design), the focus of BPM is traditionally on internal procedures and centers around the question of how a predefined outcome can be reached (“inside-out”) (Rosemann 2014). Another line of thinking takes a more external view by focusing on the resulting value propositions a process offers to customers (“outside-in”) (Rosemann 2014). Recent arguments propose that BPM practices should balance between both with an internal as well as an external view on processes (Rosemann 2014). This idea originates from and complies with the dual capability concept of organizational ambidexterity (March 1991; O'Reilly and Tushman 2013). In analogy to this concept, ambidextrous BPM has to balance between exploitative and explorative BPM activities (Rosemann 2014, 2020).

Table 1 defines central dimensions of process design and contrasts exploitative and explorative BPM. *Explorative BPM* has been defined as opportunity-driven, proactively aiming at delivering new value propositions through reengineered or new business processes (Grisold et al. 2019; Rosemann 2014). A new value proposition conveys a superior value which customers can expect when engaging with an organization (Payne et al. 2017), and business processes provide the basis for this (Dumas et al. 2018). Therefore, explorative BPM follows an outside-in logic by utilizing business and technological opportunities. This stands in contrast to *exploitative BPM* which follows an inside-out logic aiming to provide the same or enhanced value propositions by improving (i.e., incrementally changing) or reengineering (i.e., radically changing) existing business processes (Grisold et al. 2019; Rosemann 2014). Exploitative BPM has been characterized as problem-driven and reactive (Grisold et al. 2019; Rosemann 2014). A large body of BPM approaches focuses on exploitation, as the provision of new value propositions was hitherto not in the focus of BPM in research and practice (Rosemann 2020; Schmiedel and vom Brocke 2015). Following the idea of organizational ambidexterity, BPM should also consider exploration to become a key driver of corporate success (Mendling et al. 2020; Schmiedel and vom Brocke 2015). Methods are key to implementing BPM (vom Brocke et al. 2020). They define a systematic structure for performing work steps and achieving predefined goals (Braun et al. 2005). Methods feature four *attributes* (goal orientation, systematic approach, principle orientation, repeatability) and five *elements* (activities, techniques, tools, roles, output) (Braun et al. 2005; Denner et al. 2018). In the BPM context, methods are defined as sets of tools and techniques that support and enable consistent activities along the BPM lifecycle (Dumas et al. 2018).

**Table 1:** Contrasting exploitative and explorative BPM, adapted from Grisold et al. (2019)

*Three dimensions of process design*			
Trigger	Problem-driven		Opportunity-driven
Action	Improve existing process	Reengineer existing process	Create new process
Value proposition	Same value proposition	Enhanced value proposition	New value proposition

*Typical combinations for explorative and exploitative BPM*	
Exploitative BPM	(1) Problem + Improve existing process + Same value proposition
	(2) Problem + Reengineer existing process + Same value proposition
	(3) Problem + Improve existing process + Enhanced value proposition
	(4) Problem + Reengineer existing process + Enhanced value proposition
Explorative BPM	(5) Opportunity + Reengineer existing process + New value proposition
	(6) Opportunity + Create new process + New value proposition

The prevalence of an exploitative focus in BPM is reflected in the majority of BPM methods (Gross et al. 2019; Rosemann 2014; vom Brocke et al. 2020). There are a few exceptions, but arguably their focus differs. While BPR entails elements of exploration, e.g., by detecting new opportunities of emerging technologies to re-organize work (Hammer 1990; Kettinger et al. 1997), it does not provide a detailed method, resulting in support immaturity (Dumas et al. 2018). Moreover, a reengineered process, even though radically changed, may not offer a new value proposition. Various BPR case studies demonstrate the radicality of reengineered processes in practice work (Hammer 1990; Hammer and Champy 1994; Kettinger et al. 1997) but these initiatives do not imply new process outcomes, i.e., new value propositions in terms of products and services. As another example, explorative process design patterns provide guidance on how to bring new value propositions into existing processes (Rosemann 2020). Finally, product-based design aims at decomposing a product (outcome of a business process) into its (data) elements to develop an idea process design (Reijers et al. 2003). In short, existing approaches either focus on the design of new processes or the development of new value propositions. The key ambition of explorative BPM – i.e. designing new processes as well as new value propositions in a systematic way – has not been reflected in established approaches (Grisold et al. 2019; Rosemann 2014).

### Innovation Management

Research on innovation management (*IM*) aims at understanding how organizations develop innovations. It focuses on activities leading to the generation and implementation of marketable products, services, and business models (Adams et al. 2006; Tidd 2001). *Innovation*, then, refers to the development and commercialization of new ideas as key drivers of competitive advantage and corporate success (Fagerberg 2009). Hence, innovations can be novel with respect to the organization’s knowledge base and the general business environment (Damanpour 1996).

IM also covers the development of actionable advice for practice by providing tools, methods, and models to generate value (Tidd 2001). A well-known example used in fostering product innovation is the stage-gate model, covering six phases ranging from idea generation to performance realization (Cooper 2008). Focusing on the customer, another popular model is the staged service innovation model, comprising five phases from ideal screening to service launch (Song et al. 2009) and the job-centric approach proposing four steps, circling around opportunities arising from customer needs (Bettencourt et al. 2013). By contrast, the theory of inventive problem-solving (TRIZ) comprises four steps from specifying and generalizing a problem to generalizing and specifying a solution in order to foster innovation (Altshuller 2004).

What is common to all innovation processes is that they start with the recognition of *opportunities* (Adams et al. 2006). Opportunities are action possibilities related to the introduction of innovative products, services, and business models that build on changes in the business environment and creativity (Kirzner 1973; Schumpeter 1942). Changes in the *business environment* relate to the concepts of market pull and technology push (Herstatt and Lettl 2004), both being relevant opportunity sources. In order to detect new opportunities, the identification of trends at various levels can uncover hidden insights about customers’ future needs (Andreassen et al. 2015). Trends are general directions in which technology, business, culture, people, markets, or the economy are developing and changing (Kumar 2013). Trends vary in their impact and duration, while mega trends occur across regions, industries, and demographics and bring about major changes (Kumar 2013; Mason et al. 2015). To identify and consider trends, organizations must continually scan their business environment (Ortt and Smits 2006).

Besides identifying trends, *creativity* is an important driver for innovation. Creativity can be fostered by divergent and convergent thinking (Cropley 2006). *Divergent thinking* involves idea generation by making novel combinations between knowledge elements, recognizing potential associations, and transforming knowledge elements into new forms. By contrast, *convergent thinking* refers to the selection of ideas by evaluating and assessing them against certain criteria. Moreover, recent arguments stress that organizations should define their purpose – the driver underlying all business operations – to embrace new opportunities and foster innovation (Malnight et al. 2019; Mourkogiannis 2007).

### Integrating BPM and IM Methods

Following recent calls to make BPM more explorative, we seek to understand how opportunities can be identified and integrated into processes with novel value propositions. Based on the background from Sect. 2.1 and 2.2, Table 2 contrasts research on BPM and IM methods (Mendling et al. 2020). Both fields are concerned respectively with different phenomena, namely business processes and innovation outcomes. Accordingly, research outcomes have different scopes and aims. BPM is associated with problem-driven approaches, aiming to enhance existing processes on the grounds of detected shortcomings (Rosemann 2014). IM is concerned with identifying new products, services, and business models that arise from opportunities (Adams et al. 2006). Seen from this angle, IM methods may inspire explorative BPM activities.

**Table 2:** Contrasting BPM and IM methods

	BPM methods	IM methods
Key objective	How to enhance existing processes?	How to create new value?
Type of methods	Analytical	Creative
Essential approach	Problem-driven (reactively detect and resolve problems within business processes)	Opportunity-driven (proactively sense, seize, and transform opportunities)
Viewpoint	Inward-looking (changing existing business process)	Outward-looking (creating new products, services, business models)
Value proposition	Offer enhanced value propositions	Offer new value propositions
Focus	Exploitation (focus on internal problems)	Exploration (focus on opportunities)

## Research Method

**Fig. 1:**
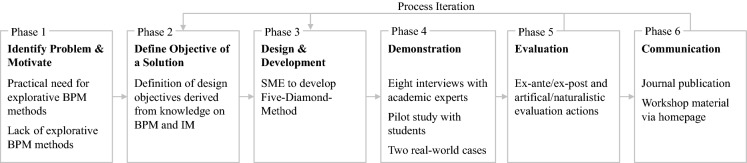
Instantiation of the DSR methodology to design the five diamond method

In this research, we seek to realize explorative BPM by systematically identifying new value propositions for business processes. Therefore, our study adopted the DSR paradigm (Gregor and Hevner 2013). Our core artifact is an explorative BPM method called the *Five Diamond Method*. In designing our method, we followed the DSR methodology (Peffers et al. 2007) comprising six phases: problem identification, definition of design objectives, design and development, demonstration, evaluation, and communication (Fig. 1).

As for *problem identification*, we justified the need for merging BPM and IM to advance explorative BPM in Sect. 1. Based on justificatory knowledge from BPM and IM, we *defined design objectives (DOs) for our solution* (Sect. 4.1). In general, DOs describe what a new artifact should look like to support solutions to problems that have not yet been addressed (Peffers et al. 2007). Hence, DOs provide guidance in the design and development phase of the DSR methodology and help to validate the artifact in the demonstration and evaluation phase.

When *designing and developing* the Five Diamond Method, we used Situational Method Engineering (SME) as artifact type-specific research method. SME distinguishes two modes, namely method configuration and method composition (Bucher et al. 2017). While method configuration refers to the adaptation of a generic method for specific situations, method composition compiles fragments from existing methods and customizes them against situational needs for achieving a certain goal (Ralyté et al. 2003). Since our goal is to develop an explorative BPM method that allows for identifying and integrating opportunities into processes with novel value propositions, we did not create an entirely new method. Rather, we synthesized existing method fragments from the BPM and IM disciplines. Furthermore, it is important to specify method requirements to clarify the situations in which a method can be used (Henderson-Sellers and Ralyté 2010). Situations are combinations of a *context type* (referring to contextual factors) and a *project type* (referring to the initial state before and a desired target state after the methods’ application) (Bucher et al. 2007). We introduce the design specification of the Five Diamond Method, including information on the context and project type as well as method fragments, in Sect. 4. More details on the context type are presented in Online Appendix 1 (available online via http://link.springer.com).

In the *demonstration and evaluation phase*, we defined an evaluation strategy that comprises evaluation activities covering an ex-ante/ex-post and an artificial/naturalistic dimension (Venable et al. 2012). The objective was to determine whether the Five Diamond Method addresses the research problem and complements existing knowledge. We performed an *ex-ante artificial evaluation* by discussing the method against literature-backed DOs and competing artifacts (Siau and Rossi 1998). To that end, we compared it with selected existing methods from BPM, i.e., BPR (Hammer and Champy 1994), product-based design (Reijers et al. 2003), and explorative process design patterns (Rosemann 2020). Furthermore, we compared it with methods from IM, i.e., the stage-gate model (Cooper 2008), staged service innovation model (Song et al. 2009), job-centric approach (Bettencourt et al. 2013), and TRIZ (Altshuller 2004). Because of the high number of available methods, we selected a sub-set of methods to conduct an in-depth comparison. We decided to include a variety of different methods in terms of several contrasting elements of BPM and IM methods, such as their key objective, type of method, essential approach, viewpoint, value proposition, and focus (Table 2). We identified these methods during the literature review for the background in Sect. 2. Finally, to ensure comparability with our method, we only considered methods from BPM and IM with an overarching perspective covering the end-to-end perspective of an improvement or innovation project, deliberately excluding specific methods with a narrow focus, such as creativity techniques. The results of the competing artifact analysis are presented in Sect. 5.1.

Moreover, we performed an *ex-ante naturalistic evaluation* through semi-structured interviews (Myers and Newman 2007) with eight industry experts. We validated the Five Diamond Method’s real-world fidelity and understandability, which are common evaluation criteria for DSR artifacts (Sonnenberg and vom Brocke 2012). In the course of this, we followed an expert sampling approach, inviting industry experts from our personal networks (Bhattacherjee 2012). An overview of the industry experts and comprehensive results are presented in Sect. 5.2. Details on the expert sampling strategy and highlights of the experts’ feedback is shown in Online Appendix 2.

Finally, we performed an *ex-post naturalistic evaluation* to validate the methods’ applicability and usefulness (Sonnenberg and vom Brocke 2012). We applied the Five Diamond Method in two phases. First, we conducted a pilot study with a group of 22 students. We used this as a first application in order to see how the method is understood and if the application goes in the intended direction. Second, we applied the method with two real-world organizations. Again, an overview of all participants and comprehensive results are reported in Sect. 5.3, with details on the application settings and results of the methods’ application provided in Online Appendix 3 and 4.

## Design Specification

### Specification of Method Requirements and Design Objectives

To ensure that the Five Diamond Method is correctly used, we recommend applying it in certain situations. This is in line with the idea of SME. We characterize these situations in terms of *context type* and *project type* (Bucher et al. 2007).

Referring to the *context type*, we use the CAMAS method to assess the context in which the Five Diamond Method is applicable (vom Brocke et al. 2020). Therefore, it facilitates the assessment of BPM methods’ applicability in terms of BPM lifecycle stages (*lifecycle dimension*), goal orientation (*goal dimension*), and three context dimensions (process, organization, and environment) of the BPM context framework (*context dimension*). A detailed assessment of the Five Diamond Method is presented in Online Appendix 1. A summary is provided in the following.

Our method can be used within the redesign stage of the BPM lifecycle (*lifecycle dimension*) to foster the exploration of business processes (*goal dimension*). Referring to the *context dimension*, our method is especially applicable for core processes to create new value proposition (*process dimension*). It is important to note that our method presupposes various stakeholders who bring in different views on emerging opportunities. In principle, our method can be used in different kinds of organizations (*organization dimension*). However, it encourages the involvement of multiple roles and stakeholders, e.g., those dealing with strategy-related matters, as well as those dealing with process-related matters. Such resources and skills are typically found in medium-sized to large organizations in the product and/or service industry. This is because medium-sized or large organizations tend to have a well-developed process orientation (Harmon and Wolf 2018; Mikalef and Krogstie 2020; Neubauer 2009), which is presupposed for the use of our method. As we will show in Sect. 5, this assumption is supported by the evaluation of our method. Finally, offering new value propositions is indispensable in competitive environments with medium or high uncertainty (*environment dimension*). One needs to take into account, however, that organizations operate in environments with different constraints in terms of laws and regulations (vom Brocke et al. 2020). Arguably, an organization that specializes in visual effects for movies has more freedom to innovate as compared to an organization that produces pharmaceutical products. Such contingencies need to be considered, especially with respect to the implementation of new solutions.

Referring to the *project type*, we assume that an organization has an established business model and existing business processes. Although the organization may be operating successfully in the market by exploiting existing processes, we suppose that the organization sets out to explore new business processes by sensing, seizing, and transforming emerging opportunities arising from customer needs and digital technologies. Hence, the need for creating new processes with novel value propositions has been recognized. In terms of the designated target state, new business processes should be proposed to create new value propositions. Accordingly, the Five Diamond Method focuses on the initial phases of the digital innovation process (Kohli and Melville 2019), comprising the idea generation and idea selection phases.

To guide the development and evaluation process of the Five Diamond Method, we derived two DOs from the problem setting specified above (Peffers et al. 2012; Sonnenberg and vom Brocke 2012) and backed them with the literature introduced in Sect. 2. Accordingly, DO.1 is derived from the definition of explorative BPM (Grisold et al. 2019; Rosemann 2014). In contrast to the goal of exploitative BPM, i.e., improving (i.e., incrementally changing) or reengineering (i.e., radically changing) existing business processes based on existing problems to provide the same or an enhanced value proposition, the ambition of the Five Diamond Method is to create new processes based on emerging opportunities to provide new value propositions for customers. To foster innovation, DO.2 addresses the respective need for actionable advice to structure the innovation process (Tidd 2001), the importance of creativity that can be fostered by divergent and convergent thinking (Cropley 2006), and the relevance of recognizing opportunities arising from new customer needs and digital technologies (Herstatt and Lettl 2004; Kirzner 1973; Schumpeter 1942). Thus, we specified that the explorative BPM method should achieve following DOs:

(DO.1) *BPM perspective*: In order to identify and integrate opportunities into new business processes, a method needs to address the exploration goal of BPM by being (a) opportunity-driven, aiming to create (b) a new process in order to provide (c) new value propositions for customer.

(DO.2) *IM perspective:* In order to identify and integrate opportunities into new business processes, an explorative BPM method needs to be (a) structured along an innovation process, (b) ensure creativity-seeking, and include (c) business and (d) technology trends as opportunity sources.

### Method Overview

**Fig. 2:**
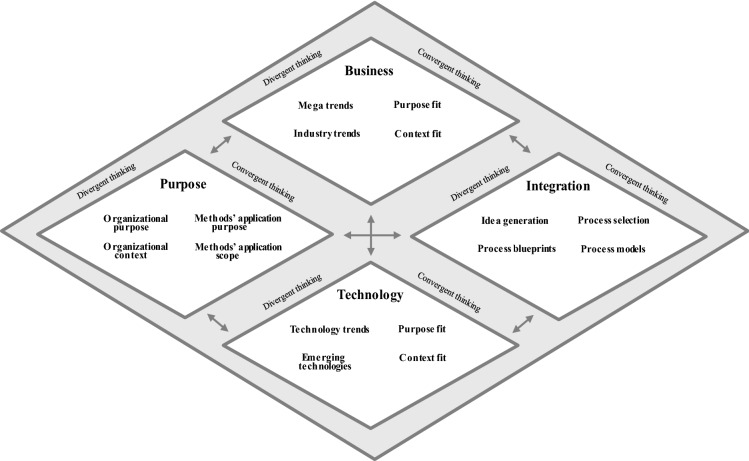
Procedure model of the five diamond method

Linking the Five Diamond Method to method attributes (goal orientation, systematic approach, principle orientation, repeatability) and elements (Sect. 2.1), the method assists organizations in identifying and integrating opportunities into new business processes to create new value propositions (*goal orientation*). Therefore, it entails various activities depicted as one overarching diamond and four underlying diamonds (Fig. 2). The four diamonds refer to (1) purpose, (2) business, (3) technology, and (4) integration. The diamond shape of these activities reflects the use of divergent and convergent thinking during the process, which are derived from IM (Sect. 2.2) (Cropley 2006). In visualizing our method and the underlying activities as diamonds, we follow popular models which have already been established (e.g., Clune and Lockrey 2014).

All activities (‘diamonds’) draw on existing knowledge from BPM and IM (Sect. 2) (*principles orientation)*. Hence, the *purpose diamond* refers to the need of gaining awareness about the purpose of the organization as well as the given context at the beginning of the innovation process (left diamond) (Malnight et al. 2019; Mourkogiannis 2007) (Sect. 2.2). To recognize emerging opportunities, trend analysis plays a crucial role during the innovation process (Ortt and Smits 2006) (Sect. 2.2). According to established concepts in the IM discipline, business and technology trends are relevant opportunity sources. Thus, the *business diamond* aims to identify opportunities related to new opportunities for generating value, e.g., through emerging customer needs (upper middle diamond). As digital technologies are important drivers for innovation (Mendling et al. 2020; Yoo et al. 2010), the *technology diamond* aims to identify opportunities for utilizing them (upper lower diamond). The *integration diamond* combines the purpose of the organization with arising opportunities to design new business processes with novel value propositions (Sect. 2.1) (right diamond). The *overarching diamond* links all underlying diamonds and provides guidance on how to execute them (*systematic approach)*.

Depending on the specific needs, an organization may choose different starting points and omit activities or the use of certain techniques. In most situations, it is useful to start with the purpose diamond. This allows participants to account for the organization’s broader context and to reflect on the strategic relevance of innovation. However, organizations have freedom in navigating through the activities. Furthermore, since the method aims to foster creativity and innovation, the application should be highly iterative. This is depicted by the bi-directional arrows between all pairs of diamonds. For example, an organization may start with the technology diamond and then proceed to the business diamond. Here, one may identify new business opportunities, which in turn can point to technology trends that have not been considered before. One logical requirement is that the method closes with the integration diamond. This is to ensure that novel and innovative ideas are being realized by means of new business processes with novel value propositions.

Figure 2 shows the iterative procedure model of the Five Diamond Method and Table 3 provides an overview of all diamonds. We introduce more details including the constitutive elements (*activities*, *techniques*, *tools*, *roles*, and *output*) for each diamond to support their execution in various contexts and among various users (*repeatability*) in Sects. 4.3 to 4.6. Table 3 stresses that the method aims to involve several stakeholders. This is to ensure that various aspects of the organization are considered during the innovation process. Like other innovation methods (e.g., design thinking), we suggest including at least one facilitator (e.g., researcher, consultant, experienced employee) who knows the method, moderates between participants and facilitates the overall procedure.

**Table 3:** Overview of the five diamond method’s elements

Activity	Technique	Tools	Roles	Output
Activity 1: Purpose diamond	*Divergent thinking* (1) Define the purpose of the organization (2) Define the organizational context of the organization *Convergent thinking* (3) Define the purpose of the method application (4) Define the scope of method application (business unit, department, etc.)	Group discussions related to organizational purpose, context and scope of method application Industry classification schemes (e.g., NACE, GICS)	BPM manager/process consultant* Senior manager** Facilitator	Defined purpose as boundary conditions for activity 2 to activity 4
Activity 2: Business diamond	*Divergent thinking* (1) Identify mega trends (2) Identify industry trends (in industry in focus and related industries) *Convergent thinking* (3) Evaluate mega and industry trends (in line with the purpose) (4) Select relevant mega and industry trends (in line with the purpose)	Multi-source research (e.g., internet, competitors, interviews, conferences, market research institutes)	BPM manager/process consultant* Business and market analyst** Business developer** Facilitator	Relevant mega and industry trends
Activity 3: Technology diamond	*Divergent thinking* (1) Identify technology trends (2) Identify existing and emerging digital technologies (in industry in focus and related industries) *Convergent thinking* (3) Evaluate technology trends and digital technologies (in line with the purpose) (4) Select relevant digital technologies (in line with the purpose)	Multi-source research (e.g., internet, competition, interviews, conferences, Gartner Hype Cycle)	BPM manager/process consultant* Technology/Digitization expert** Facilitator	Relevant technology trends and digital technologies
Activity 4: Integration diamond	*Divergent thinking* (1) Derive ideas from purpose, business, and technology diamond (2) Develop process blueprints of new processes *Convergent thinking* (3) Evaluate process blueprints (4) Select appropriate blueprints to develop new process designs	Creativity tools (e.g., brainstorming, mind-mapping) Modelling language (e.g., BPMN 2.0) Evaluation criteria (e.g. feasibility, costs, time-to-market)	BPM manager/process consultant* Innovation manager** Project portfolio manager** Senior manager** Facilitator	List of innovative process ideas New process designs

^*^BPM-related stakeholder

^**^BPM-unrelated stakeholder

### Purpose Diamond

The *purpose diamond* aims to reveal the underlying driver of organizational activities. Hence, it abstracts away from what the organization is currently doing to what drives and motivates its business. Activity 1 is carried out by BPM-related stakeholders (e.g., BPM manager, process consultant) and (senior) managers who are familiar with or even involved in the planning of strategic goals (*role*).

During the divergent phase, activity 1 requires defining the purpose and context in which the organization is operating (*technique*). Reflecting on the purpose of the organization encourages participants to reveal and discuss underlying assumptions, values, and norms of the organization and define them in the absence of their products and services (e.g., the purpose of the car manufacturer BMW is not to produce cars, i.e., specific products, but to provide mobility, i.e., abstracting away from current products/services) (Blunck 2016). Furthermore, the organization should map out the future strategy considering the context of their industry and the customers they are addressing (e.g., BMW is operating in the automotive industry) (*technique*). This can be supported by means of industry classification schemes and discussion rounds (*tool*).

The convergent phase serves to define the purpose and scope of applying the Five Diamond Method (*technique*). This is important to align expectations as well as the foci of all participants. The scope of the methods’ application refers to the entire organization or a specific unit. Accordingly, the boundary conditions for the following activities are defined (*output*).

### Business Diamond

The *business diamond* aims to identify opportunities arising from the business environment. Various BPM-related stakeholders (e.g., BPM manager, process consultant) and BPM-unrelated stakeholders are involved (e.g., business and market analysts, business developer) (*role*).

The activity starts with the divergent phase by identifying mega trends followed by industry trends (*technique*). Mega trends are global trends such as urbanization or mobility, while industry trends occur within a specific industry and reflect customer needs and/or activities by competitors (e.g., a higher demand for environmentally-friendly cars in the automotive industry). Related industries should also be considered, as they may unveil additional trends which could be transferred to the given context. Multiple sources such as internet research or market research institutes can be used to cover a broad spectrum of trends (*tool*).

Subsequently, during the convergent phase, the identified mega and industry trends are evaluated by considering the purpose defined in activity 1 (*technique*). Based on this evaluation, relevant trends for the organization at hand are selected (*output*).

### Technology Diamond

The *technology diamond* aims to capitalize on opportunities arising from digital technologies. Therefore, BPM-related stakeholders (e.g., BPM manager, process consultant) and BPM-unrelated stakeholders providing a technology perspective (e.g., technology or digitalization expert) are involved (*role*).

During the divergent phase, this activity is concerned with the identification of emerging technology trends and existing digital technologies which could be relevant for the organization (e.g., digital ecosystems that can be created around vehicles, including apps and integrated GPS-tracking systems) (*technique*). Technology trends are often associated with digital technologies as important drivers for innovation (Mendling et al. 2020), both in terms of how organizations manage their processes and serve their customers’ needs (Yoo et al. 2010). Technologies can be potentially relevant even if there are no existing applications in the organization’s industry or context (Du et al. 2019). Again, multiple sources such as internet research or the Gartner Hype Cycle can be used (*tool*).

During the convergent phase, technology trends and digital technologies are discussed in relation to the applicability to the organization (*technique*). The selection of technologies should be in line with the purpose. Relevant technologies are selected in terms of how well they fit the organization. Based on this evaluation, relevant trends for the organization are selected (*output*).

### Integration Diamond

The *integration diamond* combines identified opportunities with a BPM perspective to generate and design innovative process ideas. This is best done by BPM experts together with innovation managers, project portfolio managers, and senior managers (*role*).

During the divergent phase, this activity intends to integrate insights that have been gained in previous activities. It strives for generating innovative process ideas based on the purpose, business, and technology diamonds and independently from existing organizational constraints (e.g., processes related to car-sharing projects and initiatives to develop an infrastructure for electricity chargers) (*technique*). These process ideas can build on one or multiple opportunity sources. The idea generation can be facilitated by using creativity techniques (*tool*). In line with the definition of explorative BPM, these process ideas should offer new value proposition. To ensure a shared understanding, the generated process ideas are then translated into process blueprints (*technique*) using a process modelling language (*tool*).

During the convergent phase, the generated process blueprints are then evaluated based on selected criteria, e.g., feasibility, costs, expected value, and strategic alignment (*technique*). These criteria are then discussed in relation to organizational needs and the given context. Based on this evaluation, the most promising processes are selected. As a result, one or more process blueprints are generated to create a new process while offering a new value proposition for customers. They are thus in line with the idea of explorative BPM (*output*). Hence, the integration diamond capitalizes on the strengths that have been accumulated within the BPM discourse by explaining how novel ideas can be organized and managed (Mendling et al. 2020).

## Evaluation

### Competing Artifact Analysis

In line with our evaluation strategy (Sect. 3), we performed an *ex-ante artificial evaluation* in terms of a competing artifacts analysis. The results are shown in Table 4.

**Table 4:** Results of competing artifact analysis

Design objectives (DO)	BPM discipline				IM discipline			
	Five-Diamond-Method Business	Process Reengineering (Hammer and Champy 1994)	Product-based design (Reijers et al. 2003)	Explorative process design patterns (Rosemann 2020)	TRIZ (Altshuller 2004)	Stage-gate model (Cooper 2008)	Staged service innovation model (Song et al. 2009)	Job-centric approach (Bettencourt et al. 2013)
*Process perspective*								
(DO.1) Exploration	✓	– Exploitation	– Exploitation	✓	– Exploitation	✓	✓	✓
(DO.1a) Opportunity-driven	✓	(✓)	– Problem-driven	✓	– Problem-driven	✓	✓	✓
(DO.1b) New process	✓	– Existing process	– Existing process	– Existing process	– Existing product	– New product	– New service	– New service
(DO.1c) New value	✓	– Enhance value	– Enhance value	✓	– Enhance value	✓	✓	✓
*Innovation perspective*								
(DO.2a) Innovation process	(✓) idea generation and selection	– Principles	– Redesign process	– Patterns	– Problem-solving process	✓	✓	✓
(DO.2b) Creativity-seeking	✓	– Analytical	– Analytical	– Analytical	✓	✓	✓	✓
(DO.2c) Business trends	✓	– No trend seeking	– No trend seeking	– No trend seeking	– No trend seeking	✓	✓	✓
(DO.2d) Technology trends	✓	✓	– No trend seeking	– No trend seeking	– No trend seeking	– No trend seeking	– No trend seeking	– No trend seeking

✓ = fulfilled; (✓) = partially fulfilled; – = not fulfilled

The *competing artifact analysis* revealed that existing approaches from the BPM and IM disciplines only partially meet our DOs. Competing artifacts from the BPM discipline, BPR (Hammer and Champy 1994) and product-based design (Reijers et al. 2003), focus on exploiting existing processes by incrementally or radically changing them. Hence, they do not meet DO.1, as they are not opportunity-driven aiming to create new processes with novel value propositions. The explorative design patterns (Rosemann 2020) investigate opportunities to create new value propositions for existing processes. However, these patterns do not fulfill DO.1, as they do not initiate an innovation process, create new business processes, or include opportunity sources arising from the business environment or digital technologies. Compared to competing artifacts from the IM discipline, TRIZ (Altshuller 2004) does not address DO.2, as it focuses on solving problems rather than seeking opportunities. While the stage-gate model (Cooper 2008), the staged service innovation model (Song et al. 2009), and the job-centric approach (Bettencourt et al. 2013) seek opportunities, they focus on innovating products and services but neglect DO.2 in terms of innovating processes. Moreover, these approaches mainly focus on business opportunities but neglect technology opportunities.

The Five Diamond Method addresses both DOs. Regarding DO.1, our method is an explorative BPM method that aims to identify and integrate opportunities into new processes with novel value propositions. As for DO.2, our method structures the innovation process along four activities. It foregrounds fundamental activities of the digital innovation process (Kohli and Melville 2019); it starts with the recognition of opportunities and further focuses on idea generation and selection of innovative process ideas. However, testing and launching activities are not included. This is due to the fact that the initial, creatively intense phases, i.e., idea generation and selection, are poorly understood while the subsequent, less creatively intense phases, i.e., testing and implementation, aim to realize the benefits of selected process idea (Kohli and Melville 2019). Hence, we deliberately decided to focus on the beginning of the innovation process. We get back to this deliberate scoping decision in Sect. 6. Moreover, the method enhances creativity-seeking as it fosters divergent and convergent thinking as well as considers various opportunity sources.

Overall, the competing artifact analysis confirms that our method responds to the research question and provides advantages in relation to the DOs. This is due to the fact that the initial, creatively intense phases, i.e., idea generation and selection, are poorly understood while the subsequent, less creative intense phases, i.e., testing and implementation, aim to realize the benefits of selected process idea. We critically reflect on this decision within our competing artifact analysis in Sect. 5.1 and address the implications in Sect. 6.4.

### Expert Interviews

To complement the ex-ante artificial evaluation, we conducted an *ex-ante naturalistic evaluation* to challenge the real-world fidelity and understandability of the Five Diamond Method*.* Hence, we discussed the method with eight industry experts who are involved in BPM activities in their everyday jobs. Table 5 shows all industry experts and respective organizations that participated in the evaluation.

**Table 5:** Overview of the industry experts

ID	Current position/job title	Work experience (years)	Academic background	Industry	Employees
1	Head of Process and Change Management	> 10	Business administration	Production – Glass (2017)	
2	Innovation Manager	> 5	Information systems	Service – Insurance and financial services	7.000 (2017)
3	Management Consultant	> 18	Business administration	Service – Business Consulting	1 (2019)
4	Chief Executive Officer/Chief Disruptor	> 18	Business administration	Service – Business Consulting	3 (2019)
5	Process and Quality Manager	> 13	Business administration	Service – Communication	18,700 (2018)
6	Settlement and Collateral Management	> 2	Information systems	Service – Financial services	1100 (2019)
7	Chief Executive Officer/Process Consultant	> 24	Engineering	Service – Business Consulting	26 (2019)
8	Authorized officer/Process Consultant	> 15	Economics	Service – Business Consulting	3 (2019)

Overall, the experts affirmed the relevance of our research, as many organizations face pressures to create new processes, which in turn create new value propositions for customers. All experts appreciated the development of an explorative BPM method as a scientifically sound, yet pragmatic way to identify and integrate opportunities into business processes. The experts also identified challenges regarding the methods’ applicability. Online Appendix 2 provides an overview of the experts’ feedback and reports on how we incorporated it. Below, we present the most important results.

As for *real-world fidelity*, all industry experts confirmed that the Five Diamond Method leads to useful solutions (Sect. 4.1). They appreciated that the method distinguishes between business and technology trends as relevant opportunity sources in the digital age. The experts also acknowledged that our method allows for a flexible configuration of all diamonds. Hence, some organizations may consider all diamonds, whereas others may place more emphasis on one diamond over another. Furthermore, within each diamond, organizations can choose their appropriate level of detail by only focusing on well-known or emerging digital technologies.

As for *understandability*, the industry experts confirmed that the Five Diamond Method is comprehensible for practitioners who are typically involved in BPM or IM activities. In their view, this is supported by the consistent representation of all diamonds, each including four techniques and building on the concept of divergent and convergent thinking. Moreover, the industry experts acknowledged that all techniques are specified by respective tools and roles. This systematic structure enables the creation of new processes. However, the specification of diamonds and the appropriate level of detail might constitute a challenge. We addressed this challenge when reporting on the method application in Sect. 5.3. We provide related recommendations for application in Online Appendix 5.

### Real-World Applications

We conducted an *ex-post naturalistic evaluation* in two phases, namely (1) a pilot study with students, and (2) two real-world applications to gain experience in data collection (e.g., identifying and selecting industry and technology trends) as well as insights into the method’s applicability and usefulness.

#### Pilot Study with Students

**Fig. 3:**
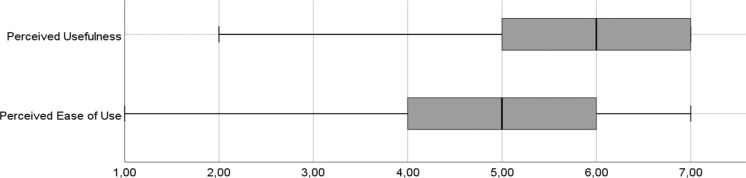
Perceived usefulness and ease of use from 1 (low) to 7 (high) (first phase of application)

In the first phase, 22 students pilot-tested the method. Dividing the students into six groups, the pilot study enabled us to evaluate how the method is understood and applied from people outside the research project. Three different real-world organizations from different industry sectors (a fashion retailer, a utility provider, and an airline) were allocated to the student groups. Each student group had the task to act as consultants and go through all diamonds of the method (within a 3-week timespan) with the aim of creating an explorative process for their respective organization. After the application, we asked all students to fill in a questionnaire in order to obtain quantitative data about perceived usefulness and ease of use (Davis 1989). These two concepts are associated with user satisfaction (Maes and Poels 2007). The students were also asked to comment on the methods’ usefulness and applicability (Sonnenberg and vom Brocke 2012). This provided additional qualitative insights. Figure 3 presents results of the evaluation. We provide details on the setting and the questionnaire in Online Appendix 3.

#### Real-World Application: Insurance Company

The first evaluation took place within a half-day workshop with an insurance company. Three innovation managers from the case organization and four co-authors participated in the workshop. The innovation managers had more than 5 years’ experience and differed in terms of personal and academic backgrounds (not restricted to solely BPM backgrounds).

The motivation behind applying the Five Diamond Method was to find innovative responses to the COVID-19 outbreak in the form of new sales processes. The focus of the workshop was opportunity-driven, as the organization aimed to integrate business and technology opportunities into new business processes. To account for the relatively short time span of the workshop, the practitioners gathered ideas in relation to the first three diamonds (purpose, business, and technology) prior to the workshop. It was found that the purpose of the insurance company is to provide “security for people”. For business trends, the practitioners translated possible scenarios of societal mega trends resulting from the COVID-19 pandemic into opportunities for the insurance sector. Over the course of the workshop, the company gathered numerous additional ideas. The technology trends were the main source of inspiration, but the participants also integrated business trends. These insights were used to generate process ideas to deliver new value to customers. We present one exemplary idea below. Detailed results are reported in Online Appendix 4.

One resulting explorative process idea evolved from integrating the business trends *Gamification* and *Connectivity* with the technology trend *Wearable Computing*. The resulting idea uses data provided by smartwatches to share and compare product-related metrics with friends through an accompanying mobile application. More specifically, the process is envisioned to enable customers to collect reward points for certain activities (e.g., a specific number of steps walked a day) which can be tracked through a smartwatch. This data can then be shared and compared with other customers of the insurance company (e.g., family or friends). The reward points can be exchanged for certain benefits. The additional values gained through this process are *Fun/Entertainment* and *Motivation*, which were not considered in any other existing process. It thus changed the previously neutral interaction with the customer to a more engaging experience. The practitioners expected that this would increase customer satisfaction, which is associated with a positive impact on customer loyalty and customer demand.

#### Real-World Application: Facility Management Company

The second real-world evaluation took place within a half-day workshop with a company that automates facilities by equipping them with sensors (e.g. smart offices). The goal of this workshop was to explore new means for applying sensor-data in space (commercial space, such as offices, or public space, such as train stations). The CEO of this company participated in this workshop alongside two co-authors and another researcher. Due to COVID-19, the workshop was held online.

The company gathered relevant knowledge about different types of sensors prior to the meeting. The purpose was defined in terms of “making space enjoyable to anyone who is inhabiting it”. The workshop started with the general idea that one can use sensor-data to inform decisions on how space can be used. For example, different sensors can be installed in a meeting room. The resulting data could be collected on platforms where different stakeholders could gain relevant information. For example, cleaning staff could be provided with information about whether or to what extent the room needs to be cleaned. Thus, the workshop was opportunity-driven in terms of new technologies (i.e., sensors) and business opportunities (i.e., platform ecosystems). Technology trends, such as person-based sensor data, environment-based data, and big data analytics, were identified as being relevant in this context. In terms of business trends, environmental sustainability and mobility were considered important.

One resulting explorative process idea is to install sensors and sell relevant information to other businesses, such as real estate agencies. This was primarily driven by the technology trend *connectivity* and the business trend *evidence-based decision making*. Sensors can be implemented in different parts of a city to measure air quality, noise exposure, and vibrations at different times of the day, week, and month. The data reflect the broader environment in which a certain object is placed. When integrated in real estate selling and renting processes, customers can gain a better idea of what it may feel like to live in a specific part of the city. This can be enhanced by measuring the same data points in the object where the customer currently lives. Furthermore, real estate agents can use these data to compare offerings in the same city across districts or inform renovation work (e.g., above-average noise levels can be compensated by means of noise insulation). Such insights can strengthen trust in estate-related information.

#### Learnings from Real-World Applications

Overall, the pilot study as well as the two real-world applications showed that the method can be applied in workshop settings. As for *applicability*, one practitioner noted, “*I liked the fact that it gave the task a structured approach*” and “*each diamond frames a different perspective while thinking about new process ideas*”. Hence, it may be appropriate to provide structure outside of this format over a longer time span. However, the practitioners articulated the concern that the brainstorming sessions during the workshop led to many ideas outside of the initially defined purpose of the application (i.e., sales processes). They asked for “more guidance and structure within each activity”, e.g., by defining the number of process ideas to be collected. They also suggested having “clear cuts” between the divergent and convergent thinking parts within each activity, e.g., by allocating pre-defined time slots. On a related note, one respondent mentioned that the level of abstraction was not clear upfront. According to him, the method allows one “*to think very big but you can also stick to a narrow focus throughout*”. In this regard, he stressed that it was important to have a facilitator who guides through the method. The practitioners also communicated that they would need more time in future applications.

As for *usefulness*, the practitioners suggested that it is useful to generate process ideas based on different activities. One practitioner commented, “*I liked that the output is a concrete process. So, it begins very abstract and ends with a concrete process*”. Similarly, the students found the method to be helpful in generating new process ideas. This can also be seen in the perceived usefulness and ease of use values (Fig. 3). The practitioners shared this view, as indicated by the following statement: “*We usually track technology trends on an ongoing basis, but not with the explicit intention to derive new processes*”. One aspect that is not covered in the method refers to the implementation of the new process ideas. This was considered an issue by the representative of the small organization (real-world application 2). Due to a lack of resources and roles, the CEO would be responsible for implementing new process ideas. This not only requires considerable effort but also poses challenges in terms of culture and organizational learning. In his view, this should be considered as well, even if informally after the workshop. We considered the feedback in our recommendations for the method’s application provided in Online Appendix 5.

## Discussion and Outlook

This study departed from the question of how we can make BPM more explorative, which was motivated by recent calls in the BPM discourse (Grisold et al. 2019; Rosemann 2020; vom Brocke et al. 2020). The underlying argument is that BPM should benefit from emerging innovation opportunities arising from business trends and digital technologies. In response, we developed and evaluated the Five Diamond Method that aims to identify and integrate various opportunity sources and translate them into new business processes with novel value propositions. In the following, we point to key contributions of our research. We acknowledge limitations and outline avenues for future research.

### Integration of Business Process Management and Innovation Management

First and foremost, our study has implications for BPM. Traditionally, BPM has been concerned with analyzing and improving existing business processes (Dumas et al. 2018). While the pioneering works of Hammer and Champy (1994) proposed that business process change should be characterized by fundamentally rethinking how work is organized in and around the organization, subsequent research has been moving towards exploitative BPM. This is reflected by the large repertoire of methods that aim to incrementally improve business process work, e.g., by increasing efficiency and effectiveness (Gross et al. 2019; vom Brocke et al. 2020). The Five Diamond Method provides a means for explorative BPM, addressing calls for new approaches to integrate innovation opportunities into new processes to offer new value propositions (Grisold et al. 2019; Rosemann 2020). This is considered particularly important in times of digital innovation where emerging technologies afford new ways to execute processes or establish new value propositions (Mendling et al. 2020). To keep pace with these dynamics, organizations not only need to enhance established processes, but also systematically explore new opportunities and integrate them into their process landscape. To that end, we adopted principles of IM to identify opportunities arising from business environments and digital technologies. Hence, our method broadens the scope of established BPM methods. It builds on conceptual claims that BPM should become more explorative (Rosemann 2014) and presents concrete steps for realizing this (Mendling et al. 2020).

### Methodological Guidance for Business Process Exploration

Our method has implications for management practices in the context of BPM. As mentioned above, BPM traditionally pursues a prescriptive research agenda, focusing on tools, methods, and models to design, improve and run business processes (Dumas et al. 2018). This is challenging in times of digitalization simply because it is impossible to predict or even anticipate emerging opportunities at speed and at scale (Benbya et al. 2020; Nambisan et al. 2017). In addition, managers are often unable to detect opportunities because they are absorbed with existing practices and logics (Grisold et al. 2020). Our method can foster business process exploration (1) by periodically screening and monitoring business and technology opportunities, and (2) by integrating them into new business processes with novel value propositions. In doing so, our method offers a means to identify and explore new configurations within the design space of business processes (Gross et al. in press). Our approach challenges dominant assumptions about process work where re-design activities are initiated after problems were detected (Table 2). Accordingly, our method provides prescriptive management advice by simultaneously considering unfolding potentials of digital technologies and business trends. As shown throughout our real-world application, our method responds to recent calls to support (process) managers and organizations in capitalizing on emerging opportunities of digital technologies (Mendling et al. 2020; Mikalef and Krogstie 2020). Our evaluation also showed that the effectiveness of our method can be increased by a facilitator, i.e., a method expert who guides participants through the overall procedure. This observation is in line with other approaches that aim to enhance creativity and innovation capabilities (e.g., design thinking). In our case, we as co-authors and method engineers facilitated the workshops.

### Methodological Integration of Digital Technologies

We also assert that this work has implications for IM. While IM has traditionally acknowledged the role of technology in innovation processes (Adams et al. 2006), recent claims suggest that digital innovation has important implications. Digital technologies are malleable and generative, hence, they enable continuous innovation (Benbya et al. 2020; Yoo et al. 2010). This is supported by recent research in the information systems field where several studies find that technologies, business models, and organizing logics are co-evolving (Sandberg et al. 2020). In light of these developments, Nambisan et al. (2017) introduce the term “digital innovation management”, proposing that IM should revisit its core assumptions. We suggest that the Five Diamond Method can contribute to this emerging discourse in two ways. First, digital IM assumes that the locus of innovation becomes more distributed across different stakeholders. Furthermore, processes and outcomes become unpredictable. Our method capitalizes on these developments. It includes various stakeholders during the method’s application, as a diverse team composition has shown to foster creativity during the idea generation (Chamorro-Premuzic 2017). Similarly, as our evaluation suggests, putting emphasis simultaneously on purpose, business, and technology enables open-ended innovation (Nambisan et al. 2017). Second, while most IM methods focus on new value generation through new products, services, and business models, our method specifies how new value can be realized through business processes. Seen from this perspective, our research also responds to calls for new approaches that integrate opportunities from digital technologies into business processes (Beverungen et al. 2020; Mendling et al. 2020).

### Limitations and Future Research

Our research comes with limitations which are related to the design of the Five Diamond Method and its evaluation. One limitation arises from assumptions underlying the method (Sect. 4). First, our method is designed for core processes. As core processes focus on creating value for external customers, we deliberately decided to investigate how explorative BPM can be implemented at the company-customer interface to create new processes. Future research may investigate how our method can be used for management and support processes.

Second, our method focuses on the initial phases of the digital innovation process (Sect. 4.1), as related activities are poorly understood (Kohli and Melville 2019). Future research should test and implement activities to realize the benefits of selected process ideas. As studies in the organizational sciences report, the implementation of new processes can initiate complex change processes that may evolve in unintended directions, e.g., processes are not fully adopted. It is important to keep in mind that new process ideas, and especially those which appear radically new for the organization, require an organization to learn new practices while unlearning previous ones (Grisold et al. 2020).

Third, the competing artifact analysis relies on a selection of BPM and IM methods. Even though relevant insights could be derived from this sample, future research may compare the Five Diamond Method with additional BPM and IM methods to further strengthen the evaluation.

Fourth, we faced a major challenge in finding the right balance between the method’s specificity and generalizability to ensure appropriate applicability, which is a widespread issue of method engineering research. To address this challenge, we applied the method with two real-world case organizations and a pilot study with 22 students. Future research should consider further organizations from various contexts to gain experience in applying the method and utilizing its potential to put explorative BPM into practice.

Fifth, our evaluation showed that our method allows organizations to “think big” in terms of new value propositions. Arguably, the implementation of such solutions poses challenges of its own. For example, the insurance company from our real-world application will need to consider important questions regarding privacy. Furthermore, we expect that various contextual factors strongly impact how well the method can be applied. These factors hinge on external factors, e.g., the legal requirements that have to be taken into account as well as internal factors, e.g. the organizational culture (Huising 2019; vom Brocke et al. 2020).

Despite these limitations, our findings show the benefits of synthesizing adjacent research streams, i.e., BPM and IM, to broaden the scope of established knowledge on BPM. We call for more cross-disciplinary research, enabling BPM to provide the guidance needed in the digital age.

## Supplementary Information

Below is the link to the electronic supplementary material.

Supplementary file1 (PDF 224 kb)
