# Supplementary material for: The Five Diamond Method for Explorative Business Process Management
Source: Bus Inf Syst Eng. 2021 Jun 8;64(2):149–66. doi: 10.1007/s12599-021-00703-1 (PMC8185488; doi:10.1007/s12599-021-00703-1)
Supplement: Supplementary file 1 — Supplementary file1 (PDF 224 kb) [file 12599_2021_703_MOESM1_ESM.pdf]

The Five Diamond Method for Explorative Business Process Management

Thomas Grisold, Steven Groß, Katharina Stelzl, Jan vom Brocke, Jan Mendling, Maximilian Röglinger, Michael Rosemann

Business & Information Systems Engineering (2021)

Appendix (available online via http://link.springer.com)

Appendix 1. Assessment of Five-Diamond-Method using the CAMAS Method

To define the context type of a method, i.e., the situations in which the method can be used, we use the CAMAS method (vom Brocke et al. 2020). According to four activities of the Assessment Process of the CAMAS method, the Five-Diamond-Method is assessed as shown in Figure A-1.

Lifecycle dimension
Improvement and innovation stage

Goal dimension		
Focus	Exploration	a
	Exploitation	na

Process dimension		
Value contribution	Core process	a
	Management process	na
	Support process	na
Repetitive-ness	Repetitive	a
	Non-repetitive	a
Knowledge intensity	Low knowledge-intensity	a
	High knowledge-intensity	a
Creativity	Low creativity	a
	High creativity	a
Inter-dependence	Low interdependence	a
	High interdependence	a
Variability	Low variability	a
	High variablity	a

Organization dimension		
Scope	Intra-organizational processes	a
	Inter-organizational processes	a
Industry	Product industry	a
	Service industry	a
	Product & service industry	a
Size	Start-up	na
	Small and medium enterprise	a
	Large organization	a
Culture	Culture highly supportive of BPM	a
	Culture non-supportive of BPM	na
Resources	Low organizational resources	na
	High organizational resources	a

Environment dimension		
Competitive-ness	Low competetive environment	a
	High competetive environment	a
Uncertainty	Low environmental uncertainty	a
	High environmental uncertainty	a

Figure A-1: Assessment of Five-Diamond-Method using the CAMAS method

Appendix 2. Overview of Expert Interviews

To evaluate the Five-Diamond-Method from an *ex-ante naturalistic perspective*, we conducted *expert interviews* (Myers and Newman 2007) following an expert sampling approach (Bhattacherjee 2012). Thus, we recruited industry experts from our personal networks that differ in their personal and academic backgrounds and cover various departments and industries. Moreover, we only included experts with more than five years of professional experience and substantial experience in BPM, IM, or business development. We conducted semi-structured interviews structured along the method's activities (Myers and Newman 2007). Each interview took about one hour and was attended by two researchers. After eight interviews, we consented that the experts' feedback was consistent and that conceptual saturation had been reached. Therefore, we did not conduct further interviews. As the consulting companies primarily advices medium-sized product organizations, they were incorporated as a multiplier despite of its small size. Table 5 within the manuscript (Section 5.2) shows all industry experts and respective organizations, Table A-1 summarizes highlights of the experts' feedback.

Table A-1: Highlights from the expert interviews

Topic	Comment	Implications
**Overview**	• Organizations must repeatedly apply the Five-Diamond-Method to ensure that once-identified trends are reviewed and updated (ID1, ID5, ID6).	• Appendix 4: Recommendation added that the Five-Diamond-Method should be applied repeatedly.
	• The order of diamonds may differ depending on the purpose of method application. Moreover, the application of the method should be iterative as business and technology trends depend on each other (ID3, ID4).	• Section 4.2: Hint added that the overarching diamond intends to execute activity 1 to 4 in the proposed order. However, different starting points can also be chosen, or activities or techniques can be omitted. Moreover, the method proposes an iterative procedure model.
	• Details for method application, e.g., workshop setting, number of trends that should be identified and selected, participants should be provided to ensure its usefulness (ID2, ID7).	• Appendix 4: Based on both real-world applications (Section 5.3) recommendations for application are proposed.
**Purpose Diamond**	• The purpose of an organization is crucial, but difficult to define (ID3, ID4, ID8).	• Section 4.3: Information added on how to define the purpose of an organization.
	• Besides the context framework additional industry classification schemes might be helpful to better define the organizational context, especially the own and relating industries (ID7).	• Section 4.2: Two industry classification schemes, i.e., GICS und NACE, are provided as potential tools to define the industry context.
**Business Diamond**	• There is a tremendous amount of industry trends that could be included. Thus, it is difficult to focus on the most relevant ones. Various evaluation criteria might also be helpful select relevant trends (ID2, ID6).	• Section 5.3 and Appendix 4: Ideas on how to focus on relevant trends (i.e., post-corona time) and evaluation criteria (e.g., relevance, enthusiasm for customers) are proposed when applying the method. Additionally, we recommend to include various stakeholder (e.g., business and market analyst) when searching for relevant business trends.

**Technology Diamond**	• When identifying digital technologies, it is important that some organizations might only focus on emerging technologies to be a first mover, while other organizations might focus on more established ones (ID1, ID5).	• Section 4.5: Hint added that the divergent thinking phase covers the collection of existing and emerging digital technologies.
**Integration Diamond**	• Generating new process ideas can not only be based on business and technology trends, this can be also done by just using the defined organizational purpose (ID3).	• Section 4.6: Hint added that new process ideas can build the purpose, business, and technology diamond as well as combinations of these.

Appendix 3. Overview Application Setting with Students

To evaluate the Five-Diamond-Method from an *ex-post naturalistic perspective*, we first taught the method to 22 bachelor students (enrolled in a business or business law major) which chose a specialization in BPM. In four sessions which lasted three hours each, the students got to know the Five-Diamond-Method and its theoretical foundation. The students then formed groups and applied the Five-Diamond-Method on case organizations they were assigned to. The students were asked to identify their organization's purpose and to justify necessary assumptions they made. The students had three weeks to apply the method on the case organization and to submit a written report.

After the students have applied the method and submitted the report, the students were asked to anonymously fill out an online questionnaire, in which 18 students participated. Through this survey, quantitative data about the perceived usefulness and ease of use could be gathered. For this, we adjusted the proposed items from (Davis 1989), with four items for each construct, e.g., "I find the Five-Diamond-Method useful for identifying innovation opportunities in business processes" for perceived usefulness. The participants were able to respond on a 7-point Likert scale with the extremes being labeled "strongly disagree" and "strongly disagree". The comprehensive results are shown in Figure 3 (manuscript).

Perceived Usefulness:

- Using the 5-Diamond method would improve my performance in identifying innovation opportunities in business processes
- Using the 5-Diamond method would improve my productivity in identifying innovation opportunities in business processes
- Using 5-Diamond method would enhance my effectiveness in identifying innovation opportunities in business processes
- I find the 5-Diamond method useful for identifying innovation opportunities in business processes

Perceived Ease of Use:

- Learning to use the 5-Diamond method would be easy for me
- I would find it easy to deploy the 5-Diamond method to do what I want it to do
- It would be easy for me to become skillful in the use of the 5-Diamond method
- I would find the 5-Diamond method easy to use

Appendix 4. Overview of Workshop Setting with Case Companies

To evaluate the Five-Diamond-Method from an *ex-post naturalistic perspective*, we applied the Five-Diamond-Method within two case organizations. The results for the insurance company are shown in Table A-2, for the facility management company in Table A-3.

Table A-2. Results of applying the Five-Diamond-Method's at an insurance company

Activity	Technique	Tools	Roles	Output
**Activity 1: Define purpose, context, and scope of method application**	*Divergent thinking*<br>(1) Define the purpose of the organization<br>(2) Define the organizational context of the organization<br>*Convergent thinking*<br>(3) Define the purpose of the method application<br>(4) Define the scope of method application (business unit, department, etc.)	- Industry classification scheme GICS	- Three innovation managers**<br>- Four BPM researchers*	*Divergent thinking*<br>(1) "Providing security"<br>(2) Insurance company (40 – Financials, 4030 – Insurance)<br>*Convergent thinking*<br>(3) Identify new processes to address opportunities of a post-corona time<br>(4) Sales department
**Activity 2: Analyze and evaluate mega and industry trends**	*Divergent thinking*<br>(1) Identify mega trends<br>(2) Identify industry trends (in industry in focus and related industries)<br>*Convergent thinking*<br>(3) Evaluate mega and industry trends (in line with the purpose)<br>(4) Select relevant mega and industry trends (in line with the purpose)	- Internet research		*Divergent thinking*<br>(1) Globalization, individualization, sharing economy, digitalization, COVID-19 pandemic<br>(2) Life insurance analytics, digital interaction, micro mobility<br>*Convergent thinking*<br>(3) Evaluation based on purpose of method application, necessity, and urgency<br>(4) COVID-19 pandemic
**Activity 3: Analyze and evaluate technology trends and digital technologies**	*Divergent thinking*<br>(1) Identify technology trends<br>(2) Identify existing and emerging digital technologies (in industry in focus and related industries)<br>*Convergent thinking*<br>(3) Evaluate technology trends and digital technologies (in line with the purpose)<br>(4) Select relevant digital technologies (in line with the purpose)	- Internet research		*Divergent thinking*<br>(1) Predictive Analytics, Social Media Data, Internet of Things, Customer Relationship Management Systems, Chatbots, Voice-driven sales apps, Sensors, Wearables, Process Mining, Mobile applications<br>(2) Artificial Intelligence, Machine Learning, Blockchain/Smart contracts<br>*Convergent thinking*<br>(3) Evaluation based on relevance and enthusiasm for customers<br>(4) Predictive Analytics, Social Media Data, Internet of Things, Customer Relationship Management Systems, Chatbots, Wearables, Process Mining, Mobile applications
**Activity 4: Identify and select process innovation ideas**	*Divergent thinking*<br>(1) Derive ideas from purpose, business, and technology diamond<br>(2) Develop process blueprints of new processes<br>*Convergent thinking*<br>(3) Evaluate process blueprints<br>(4) Select appropriate blueprints to develop new process designs	- Brainstorming<br>- Evaluation criteria		*Divergent thinking*<br>(1) Eleven ideas for new processes and services<br>(2) High-level process blueprints for each idea<br>*Convergent thinking*<br>(3) Evaluation based on feasibility, testability, time-to-market, costs, experience<br>(4) Selected top 3 ideas and designed respective process models

* BPM-related stakeholder, ** BPM-unrelated stakeholder

Table A-3. Results of applying the Five-Diamond-Method's at facility management company

Activity	Technique	Tools	Roles	Output
**Activity 1: Define purpose, context, and scope of method application**	*Divergent thinking*<br>(1) Define the purpose of the organization<br>(2) Define the organizational context of the organization<br>*Convergent thinking*<br>(3) Define the purpose of the method application<br>(4) Define the scope of method application (business unit, department, etc.)			*Divergent thinking*<br>(1) "Making space enjoyable"<br>(2) Facility management<br>*Convergent thinking*<br>(3) Identify new processes to address opportunities to use sensor data<br>(4) New business opportunities
**Activity 2: Analyze and evaluate mega and industry trends**	*Divergent thinking*<br>(1) Identify mega trends<br>(2) Identify industry trends (in industry in focus and related industries)<br>*Convergent thinking*<br>(3) Evaluate mega and industry trends (in line with the purpose)<br>(4) Select relevant mega and industry trends (in line with the purpose)	- Internet research	- CEO*	*Divergent thinking*<br>(1) Connectivity, Covid-19, transparency, smart mobility, environmental protection<br>(2) Smart home, sensor-based decision making, physical to virtual meetings<br>*Convergent thinking*<br>(3) Evaluation based on purpose of method application, necessity, and urgency<br>(4) Transparency enabled through data
**Activity 3: Analyze and evaluate technology trends and digital technologies**	*Divergent thinking*<br>(1) Identify technology trends<br>(2) Identify existing and emerging digital technologies (in industry in focus and related industries)<br>*Convergent thinking*<br>(3) Evaluate technology trends and digital technologies (in line with the purpose)<br>(4) Select relevant digital technologies (in line with the purpose)	- Internet research		*Divergent thinking*<br>(1) Big data analytics, person-related data, environment-related data<br>(2) Smart home<br>*Convergent thinking*<br>(3) Evaluation based on business opportunities<br>(4) Internet of Things
**Activity 4: Identify and select process innovation ideas**	*Divergent thinking*<br>(1) Derive ideas from purpose, business, and technology diamond<br>(2) Develop process blueprints of new processes<br>*Convergent thinking*<br>(3) Evaluate process blueprints<br>(4) Select appropriate blueprints to develop new process designs	- Brainstorming<br>- Evaluation criteria		*Divergent thinking*<br>(1) Several ideas for new processes and services<br>(2) High-level process descriptions for each idea<br>*Convergent thinking*<br>(3) Evaluation based on relevance, feasibility, customer experience<br>(4) Selected top 2 ideas – currently being modelled

* BPM-related stakeholder, ** BPM-unrelated stakeholder

Appendix 5. Learnings and Recommendations for Application

Based on both applications, we provide some recommendation for applying the Five-Diamond-Method in the following.

- We recommend including different roles within the organization to include and account for different perspectives during the divergent and convergent thinking phases of the different activities. For instance, it is beneficial to include technology experts that are knowledgeable about the organization's technical infrastructure in order to get instant feedback about the technical feasibility. Alternatively, as seen real-world application 2, one can also involve one key stakeholder who is knowledgeable about business-related matters (e.g. strategy and vision) as well as the operational details (e.g. process designs). In such cases, however, it is important to moderate the discussion in a way that the participant has time and space to slip into the respective role.
- Applying the Five-Diamond-Method within a half-day workshop was challenging from a time perspective. While we recommend that the different stakeholders prepare individually by identifying appropriate trends beforehand, it is still advised to go through each diamond in the course of the workshop in order to agree on its relevance and impact before generating and evaluating innovative process ideas. Thus, we recommend at least one full day for conducting the workshop. Furthermore, following the feedback of one of the participants, it can be beneficial for the organization to have follow-up reflection opportunities. This can be important as the implementation of new processes can pose organizational challenges.
- Regarding the sequence of activities, we realized that there are many iterations between the last three diamonds (business, technology, and integration). We think this is beneficial, as the output of one diamond can trigger innovative process ideas in another. Similar to creative techniques like design thinking, iterations through the activities can be useful as it promotes learning (Plattner et al. 2014). To account for the specifies of a workshop, the Five-Diamond-Method does not provide a recommendation regarding the time spend on each diamond or the amount of trends/process ideas to be collected. However, we recommend specifying these parameters in the beginning of a workshop in order to clarify and align expectations of the participants.
- The creative nature of the workshop makes it also possible to generate process or purpose-unrelated ideas. We thus recommend to continuously check the process focus is still maintained, especially within the integration diamond. Following the comment of one participant, it is important to involve a moderator who guides the process.

References

Bhattacherjee A (2012) Social science research: principles, methods, and practices, 2nd edn. CreateSpace Independent Publishing Platform, Tampa

Davis FD (1989) Perceived usefulness, perceived ease of use, and user acceptance of information technology. MIS Q 13:319

Myers MD, Newman M (2007) The qualitative interview in IS research: examining the craft. Inf Organ 17:2–26

vom Brocke J, Denner M-S, Schmiedel T, Stelzl K, Röglinger M, Wehking C (2020) Context-aware business process management method assessment and selection. Bus Inf Syst Eng, in press. https://doi.org/10.1007/s12599-021-00685-0
